# Synthesis of Highly-Dispersed Graphene Oxide Nanoribbons–Functionalized Carbon Nanotubes–Graphene Oxide (GNFG) Complex and Its Application in Enhancing the Mechanical Properties of Cementitious Composites

**DOI:** 10.3390/nano11071669

**Published:** 2021-06-25

**Authors:** Peiqi Li, Junxing Liu, Sungwun Her, Erfan Zal Nezhad, Seungmin Lim, Sungchul Bae

**Affiliations:** 1 Department of Architectural Engineering, Hanyang University, Seoul 04763, Korea; lipeiqi1995@hanyang.ac.kr (P.L.); liujx128119@hanyang.ac.kr (J.L.); sung0@hanyang.ac.kr (S.H.); 2 Department of Biomedical Engineering, University of Texas, San Antonio, TX 78249, USA; erfan.zalnezhad@utsa.edu; 3 Department of Architecture, Kangwon National University, Chuncheon 24341, Korea; smlim@kangwon.ac.kr

**Keywords:** graphene oxide nanoribbons, graphene oxide, functionalized carbon nanotubes, cement paste, mechanical properties

## Abstract

In this study, a graphene oxide nanoribbons–functionalized carbon nanotubes–graphene oxide (GNFG) complex was hydrothermally synthesized as a nanomaterial for reinforcing cementitious composites, using a modified Hummers’ method. Three types of components existed in the GNFG: Type I, the functionalized carbon nanotubes–graphene oxide nanoribbons (FCNTs–GNR); and types II and III are graphene oxide (GO) and functionalized carbon nanotubes (FCNTs), respectively, which exist independently. The dispersivity of GNFG and its effects on the mechanical properties, hydration process, and microstructures of cement pastes were evaluated, and the results were compared with those using cement pastes incorporating other typical carbon nanomaterials. The results demonstrated that dispersion of GNFG in aqueous solutions was superior to that of the CNTs, FCNTs, and GO/FCNTs mixture. Furthermore, the highly-dispersed GNFG (0.05 wt.%) improved the mechanical properties of the cement paste after 28 days of hydration and promoted the hydration of cement compared to CNTs, GO, and GO/FCNTs mixture (0.05 wt.%). The results in this study validated the feasibility of using GNFG with enhanced dispersion as a new nano-reinforcing agent for various cementitious systems.

## 1. Introduction

Since the development of Portland cement in 1824, it has gradually become the most extensively used building material worldwide due to its excellent performance and low cost [1]. Portland cement is usually applied as a binder to form cementitious materials, including concrete, along with other aggregates (sand and gravel). However, owing to the brittleness and lack of flexural/tensile strength of cementitious materials, many studies have focused on improving its mechanical strength. The typical method for improving the flexural/tensile strength of cementitious materials is to employ fibers such as polypropylene fiber, carbon fiber, steel fiber, and glass fiber as reinforcing agents [2,3,4,5,6]. Xue et al. [7] and Cao et al. [8] reported that the fiber exhibited various reinforcement effects on cementitious materials due to their different types and lengths. Although these fibers can improve the tensile strength and toughness of cementitious materials, delaying the transformation of microcracks into microforms, they cannot constrain the development of microcracks [9]. Nanomaterials, such as nano-silica, nano-titanium dioxide (TiO_2_), carbon nanotubes (CNTs), and graphene oxide (GO), have also been found to improve the mechanical properties of cementitious materials and hinder the spread of microcracks [10]. Among these materials, carbon nanomaterials, represented by CNTs and GO, are favored owing to their excellent mechanical properties [11,12].

CNTs, developed by Iijima in 1991 [13], are an allotropic form of carbon with unique characteristics that make them suitable reinforcing agents in various fields [14]. The carbon atoms of the tubes are sp^2^ hybridized and combine through carbon-carbon bonds (σ bond) to form a hexagonal honeycomb structure [15]. This structure provides the unique mechanical properties of CNTs. The tensile strength and Young’s modulus of CNTs exceed 60 GPa [16] and 1.2 TPa [17], respectively. Therefore, CNTs are considered to be an ideal nanomaterial and one of the most promising nanomaterials in the 21st century [18]. However, because of the strong van der Waals forces between the CNTs and the absence of hydrophilic functional groups on the surface, it is difficult to uniformly disperse CNTs in aqueous solutions. The precondition of using CNTs as a reinforcing material in cement-based materials is uniform dispersion. Three methods are currently used to disperse CNTs: ultrasonication treatment, use of surfactants, and functionalization of the CNTs [19,20,21]. Xu et al. [22] reported that CNTs could reduce the porosity of cement by filling the pores in the cement matrix, and found that the flexural strength of the cement paste increased by the addition of CNTs (0.05 wt.%) that sonication and surfactants were used.

In recent years, functionalized CNTs (FCNTs) have gradually attracted attention. The purpose of functionalizing the CNTs is to address the poor dispersion of pure CNTs [23]. CNTs can be functionalized using chemical (covalent) and physical (non-covalent) methods [24]. Generally, CNTs have several inherent structural defects. During chemical functionalization, oxygen-containing functional groups are inserted at these defect sites by damaging inherent defects using strong acids [25]. This method is easy to achieve in the laboratory; thus, in this study, we focus on the chemical method. Furthermore, Mousavi et al. [26] demonstrated that the ultrasonic treatment can increase the dispersion of FCNTs in aqueous solutions. When the FCNTs content was 0.05 wt.%, the compressive and flexural strengths of cement paste increased by 26.6% and 3.2%, respectively.

As a two-dimensional (2D) carbon nanomaterial, GO has also received widespread attention from researchers. It is a hydrophilic material with a variety of oxygen-containing functional groups (carboxyl and hydroxyl); thus, GO disperses better in aqueous solutions and is more uniformly distributed in the cement matrix than CNTs and FCNTs [27]. The 2D structure of GO confers excellent mechanical properties, which are superior to nano-fibrillar cellulose, nano calcium carbonate, and nano-clay [28]. Specifically, the elastic modulus and fracture toughness of GO are 32 GPa and 120 MPa, respectively [29]. Peng et al. [30] demonstrated that GO can improve the microscopic morphology of cement hydration products, thereby increasing the strength of hardened cement paste. When the water-to-cement ratio (w/c) was 0.35, the incorporation of 0.03 wt.% GO increased the flexural strength of the cement mortar by 21.86%. Pan et al. [31] also reported that GO interacted strongly with the cement matrix due to the wrinkled surface structure that provided more nucleation sites for cement hydration, enhancing the mechanical properties of the cement paste. An et al. [32] reported similar results: the large specific surface area of GO enhanced the nucleation effect and promoted hydration. After 28 days of curing, cement paste containing 0.05 wt.% GO exhibited increases in the compressive strength and flexural strength by 25.5% and 37.3%, respectively.

As research on the enhancing effect of single carbon nanomaterial in cement-based materials has proceeded, the synergistic effect that may exist between multiple nanomaterials has entered the vision of researchers [33]. Many studies have demonstrated the enhancing effects of CNTs or GO on cementitious materials; however, only a few studies have demonstrated the co-effect of CNTs and GO on the mechanical properties of cement paste. Lu et al. [34] found that the electrostatic repulsion between CNTs and GO in a solution can overcome the van der Waals force between CNTs, thus improve the dispersion of CNTs. Moreover, when the w/c ratio was 0.4, the flexural and compressive strengths of the cement paste containing 0.025 wt.% CNTs and 0.025 wt.% GO (total content of 0.05 wt.%) were increased by 21.13% and 24.21%, respectively. These increases were greater than those obtained from the addition of 0.05 wt.% CNTs (flexural: 10.14%, compressive: 6.40%) or 0.05 wt.% GO (flexural: 16.20%, compressive: 11.05%). Zhou et al. [35] also reported that GO/CNTs could form a relatively stable dispersion system; because of the filling effect of GO/CNTs, the porosity was reduced and the mechanical strength of the cement paste increased. Furthermore, Kaury et al. [36] reported that the dispersion of FCNTs and GO was improved after mixing owing to the co-effect, and the strength of mortars can be significantly improved when using this mixture compared to adding either FCNTs or GO. Although this synergistic effect can appropriately improve the poor dispersion of CNTs, the results are still not satisfying.

Herein, as a new type of nano-reinforcing agent in cement-based materials, highly dispersed graphene oxide nanoribbons (GNR)-functionalized carbon nanotubes (FCNTs)-graphene oxide (GO), GNFG complex was synthesized, which has good dispersion and also improves the mechanical properties of cementitious materials. The structure, functional groups, and dispersibility of GNFG were investigated and compared to those of CNTs, FCNTs, GO, and GO/FCNTs mixture. The morphologies and chemical bonds of the nanomaterials were characterized by transmission electron microscopy (TEM) and Fourier transform infrared spectroscopy (FTIR), respectively. X-ray diffraction (XRD) was employed to characterize the crystal structure of the nanomaterials and to qualitatively analyze the cement hydration products. The dispersion of these nanomaterials was tested by ultraviolet–visible (UV–vis) spectroscopy. To confirm the effect of GNFG in the cement-based materials, the hydration products, microstructure, and hydration kinetics of the cement paste with GNFG were investigated via thermogravimetric (TG) and differential thermogravimetric (DTG) analysis, scanning electron microscopy (SEM), and isothermal conduction calorimetry, respectively.

## 2. Experimental Procedure

### 2.1. Materials

Ordinary Portland cement (OPC) CEM I 42.5R provided by Sungshin Co. Ltd. (Seoul, Korea) was used as the binder material to prepare the cement paste. Multiwalled CNTs were purchased from Hengqiu Technology (Suzhou, China). Graphite flake powders were procured from Alfa Aesar (Haverhill, MA, USA). The chemical composition of OPC and the physical properties of the CNTs and graphite are shown in Table 1, Table 2 and Table 3, respectively. To disperse the nanomaterials in the cement matrix, a polycarboxylate superplasticizer (SP, ExCon SP20, Buildex Co., Ltd., Cheonan-si, Chungcheongnam-do, Korea.) was used, and its properties are listed in Table 4.

### 2.2. Preparation of GNFG, GO, FCNTs, and GO/FCNTs Mixture

#### 2.2.1. Synthesis of GNFG and GO

In 1958, Hummers et al. described a method to synthesize GO using a mixture of sodium nitrate (NaNO_3_), potassium permanganate (KMnO_4_), and concentrated sulfuric acid (H_2_SO_4_), which is also known as Hummers’ method [37]. Although Hummers’ method has been used widely to synthesize GO [38], it still suffers from several drawbacks, including the generation of toxic gases (NO_2_ and N_2_O_4_), nitrate residues, and low yields [27]. The Hummers’ method has been modified in the past 20 years to address these problems [39]. Currently, most researchers utilize modified Hummers’ method to synthesize GO, wherein the proportions of the experimental materials and process are changed [40].

**Figure 1:**
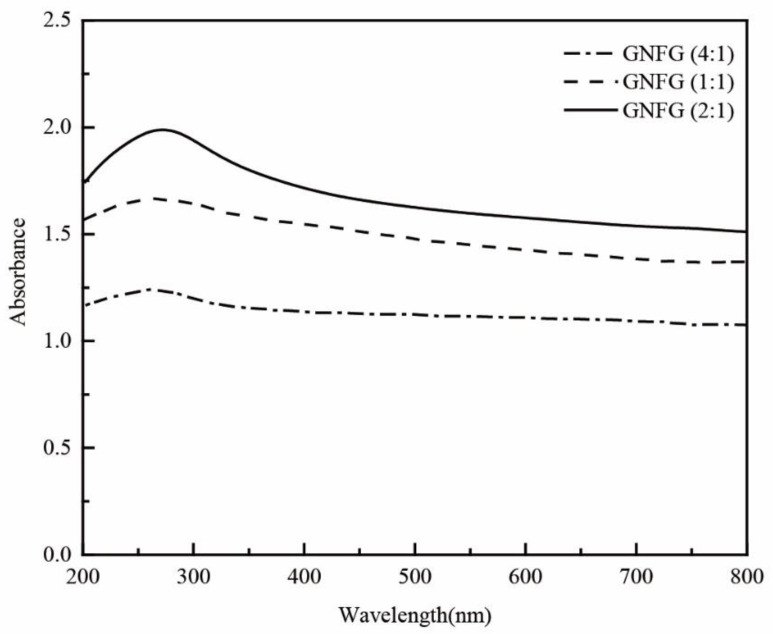
UV–vis spectra of GNFG synthesized using different ratios of graphite to CNTs.

GNFG was also synthesized using the modified Hummers’ method. First, a combined mass of 1 g of graphite and CNTs (mass ratio of graphite: CNTs = 2:1) and 0.65 g of NaNO_3_ were added to a beaker and mixed. The beaker was kept in an ice-water bath below 5 °C. Subsequently, 30 mL of H_2_SO_4_ (95%) was transferred to the beaker and the mixture was stirred. After 3 min of stirring, KMnO_4_ (3.9 g) was gradually added to the beaker and the mixture was stirred with a magnetic stirrer for 2 h. Then, the beaker was transferred to a water bath (35 °C) and kept in the water bath for 30 min. Next, 50 mL of distilled water was added dropwise to the mixture and the mixture was stirred for 30 min. The temperature of the water bath was increased to 98 °C and maintained for 20 min. Finally, a 30% H_2_O_2_ solution (5 mL) was added dropwise to terminate the oxidation reaction. The mixture was filtered, and the remaining metal ions were removed by adding a 10% HCl aqueous solution dropwise. Then, the solution was washed thoroughly with distilled water until the pH was approximately 7. Finally, the filtered solid was dissolved in distilled water, sonicated for 30 min, filtered again, and dried. A similar procedure was followed to synthesize GO using 1 g of graphite powder as the raw material. The GNFG and GO powders were obtained by drying in an oven at 60 °C for 24 h. In the preliminary experiment, the GNFG with the different mass ratio of graphite to CNTs (1:1, 2:1, and 4:1) were synthesized and their dispersity in aqueous solutions tested, as presented in Figure 1. GNFG with a mass ratio of 2:1 (graphite: CNTs) exhibited the best dispersion in the aqueous solution. Therefore, we used the GNFG with graphite and CNTs mass ratio of 2:1 for all measurements in this study.

#### 2.2.2. Functionalization of the CNTs

Many methods of functionalizing CNTs have been introduced in previous studies [41,42,43]. In our experiments, a relatively safe and simple method was chosen, as follows: H_2_O_2_ (50 mL) was slowly added to 150 mL of H_2_SO_4_ (95%) to obtain a piranha solution; the mixture was left to cool and then used. Next, 0.3 g of CNTs were added to the piranha solution and the mixture was stirred for 30 min. The mixed solution was then sonicated for 1 h. After the ultrasonic treatment was completed, the solution was diluted with distilled water, filtered, and repeatedly washed to reach a pH of approximately 7. Finally, The FCNTs powder was obtained by drying the filtered solid in an oven at 60 °C for 24 h.

#### 2.2.3. Mixing of GO/FCNTs

In order to compare with GNFG, the GO/FCNTs mixture was also prepared. We added 0.6 g of GO and 0.3 g of FCNTs (mass ratio of 2:1) to an aqueous solution and sonicated it for 15 min to ensure uniform mixing of GO and FCNTs. Then the mixture was filtered and the residue dried. Finally, a mixture of GO and FCNTs was obtained.

### 2.3. Characterization of the GRAPHITE, GO, CNTs, FCNTs, GO/FCNTs Mixture, and GNFG

The morphologies of graphite, GO, CNTs, FCNTs, GO/FCNTs mixture, and GNFG were determined using TEM (JEOL, JEM-2100F, Tokyo, Japan) with an electron source operating at an accelerating voltage of 220 kV. To prepare the samples, first, appropriate amounts of the graphite, GO, CNTs, FCNTs, GO/FCNTs mixture, and GNFG were dissolved in ethanol and ultrasonically dispersed for 15 min (Qsonica, Q700 Sonicator, Newtown, CT, USA, 20 kHz, Amplitude: 50%) [34]. Next, the dispersions of graphite, GO, CNTs, FCNTs, GO/FCNTs mixture, and GNFG were placed on a holey carbon film on 400 mesh Cu grids. Finally, the Cu grids were dried in an oven at 65 °C for 24 h.

XRD analysis was performed to study the crystalline structure of the graphite, GO, CNTs, FCNTs, GO/FCNTs mixture, and GNFG using a Bruker D2 X-ray diffractometer (λ = 1.54 Å) in the scattering (2θ) range of 5–60° with a step time of 1.5 s and step size of 0.01°. Phase identification was conducted using the DIFFRAC.EVA software. To evaluate the chemical bonds and functional groups of the graphite, GO, CNTs, FCNTs, GO/FCNTs mixture, and GNFG, FTIR was performed using a FTIR spectrometer (Spectrum Two FTIR spectrometer, PerkinElmer, Waltham, MA, USA) in the wavenumber range of 500 to 4000 cm^−1^.

A UV–vis absorption spectrophotometer (GENESYS 180, Thermo Fisher Scientific, Waltham, MA, USA) was used to measure the dispersion of the GO, CNTs, FCNTs, GO/FCNTs mixture, and GNFG in aqueous solutions. Before the test, 0.05 g of different nanomaterials were added to 30 mL of distilled water. The concentration of the test solution was the same as that of the solution added to the cement paste, i.e., 1.66 mg/mL, followed by 15 min of sonication with a sonicator (Qsonica, Q700 Sonicator) at an amplitude of 50%. As a rule, in the absorption UV–vis spectrum, the absorption is proportional to the concentration, according to the Beer–Lambert law [44]. However, only the low concentration of the solution obeys the Beer–Lambert Law [45]. Therefore, the solutions after ultrasound treatment were diluted to 0.1 mg/mL [46]. SP was not added to the solutions for this test to exclude the effect of SP on the dispersion of the nanomaterials.

An important aspect of this study is to investigate the role of highly dispersed GNFG in the enhancement of cement properties. Several previous studies have extensively compared the roles of GO, CNTs, FCNTs, and GO/FCNTs mixtures in enhancing the properties of cement [34,47,48,49,50]. However, the structure of FCNTs can be damaged by chemical treatment resulting in shorter lengths, smaller diameters, and rough surfaces. Therefore, the mechanical properties of pure CNTs are better than those of FCNTs [51]. Based on the above, in this study, the GO, CNTs, and GO/FCNTs mixture were compared with GNFG.

### 2.4. Preparation of the GO, CNTs, GO/FCNTs Mixture, and GNFG Cement Pastes

Cement paste containing 0.025 wt.% CNTs/0.025 wt.% GO composites can significantly improve various properties of the cement paste [34]. Therefore, the ratio of nanomaterials selected for this study was also 0.05% of the weight of cement, and the w/c ratio was 0.3. The solution used to prepare specimens was obtained by adding 0.05 g of GO, CNTs, GO/FCNTs mixture, and GNFG to 30 g of distilled water. This solution was treated with ultrasonic waves to uniformly disperse the nanomaterials in water. However, if the energy of ultrasonic waves is too low, the dispersion will be non-uniform. In contrast, if the energy of the ultrasound is too high, the structure of the material will be damaged [52]. Hence, to ensure the same experimental conditions, four different nanomaterials were sonicated (Qsonica, Q700 Sonicator) using the same processing time and amplitude, which were 15 min and 50%, respectively. In addition, the beaker with the solution was placed in an ice-water bath during the sonication process to prevent the solution temperature from increasing during continuous sonication. Five specimens were prepared as shown in Table 5. For each specimen, SP was added at a 0.1 wt.% dosage based on the cement weight.

Usually, SP is added to cement paste for two reasons. Firstly, SP is added to improve the workability of the cement paste, since the w/c ratio was chosen to be small and the addition of nanomaterials will cause the workability of the cement paste to deteriorate [53,54]. Secondly, SP is used as a surfactant to disperse the nanomaterials in the cement matrix [53]. Although these nanomaterials can be relatively stable dispersed in aqueous solutions after sonication, previous studies have shown that, in the alkaline environment of the cement matrix, the dispersion of CNTs and GO will be reduced, causing agglomeration [55,56]. S. Chuah [55] reported that premixing SP with cement reduces the alkalinity of the cement matrix in the early stages of cement hydration, as well as reducing the effects of alkaline conditions on dispersion. Thus, in this work, cement (100 g) and 0.1 g SP were pre-mixed to obtain a uniform mixture. Then the GO, CNTs, GO/FCNTs mixture, and GNFG solutions were mixed with the cement and SP mixture using a paste mixer (Malcom, SPS–1, Tokyo, Japan) for 10 min, respectively. Subsequently, the fresh cement paste was placed into compressive strength molds (5 × 5 × 10 mm^3^) and splitting tensile strength molds (Φ10 × 20 mm^2^) and cured for 24 h. After 24 h, the specimens were demolded and stored in a constant temperature-humidity curing cabinet; the curing temperature and humidity were 25 °C and 65%, respectively.

### 2.5. Compressive and Splitting Tensile Strength Tests

**Figure 2:**
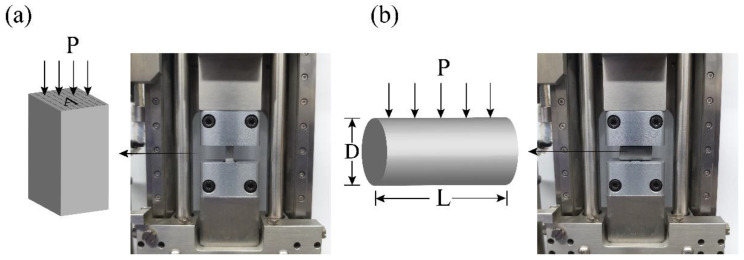
Schematics of strength tests: (**a**) compressive strength and (**b**) splitting tensile strength. Where: (**a**) F = P/A, (**b**) T = 2P/πLD; F: Compressive strength (MPa); T: Splitting tensile strength (MPa); P: Maximum load applied to the specimen (N); A: Cross-sectional area of the specimen (mm^2^); L: Length of specimen (mm); D: Diameter of specimen (mm).

To observe the variation of the mechanical properties of the cement paste with hydration time, the compressive and splitting tensile strength of the specimens were tested after curing 1, 3, 7, and 28 days, respectively [57]. The compressive and splitting tensile strengths of the hydrated cement paste were measured using a micro-compressive machine (Deben, Micro-Compressive Machine, Edmunds, Suffolk, UK) [58]. Figure 2 shows a schematic of the compressive and splitting tensile strength tests [57,59]. The sizes of the samples used for the compressive and splitting tensile strength tests were 5 × 5 × 10 mm^3^ and Φ10 × 20 mm^2^, respectively.

### 2.6. Microstructure and Mineral Analysis

The microstructures of the fracture surfaces of the specimens after curing for 28 days were analyzed by SEM (Thermo Fisher Scientific, Verios G4, Waltham, MA, USA). XRD (Bruker, D2 PHASER, Billerica, MA, USA) and TG analysis (HITACHI, STA7200 Simultaneous Thermogravimetric Analyzer, Tokyo, Japan) were used to study the cement hydration products after curing for 1, 3, 7, and 28 days. The TG experiments were conducted in a temperature range of 20 to 1000 °C with a heating rate of 10 °C/min, under an N_2_ atmosphere with a flow rate of 200 mL/min.

### 2.7. Heat of Hydration

Isothermal conduction calorimetry (TA instrument, TAM-air, New Castle, DE, USA) was conducted to evaluate the effects of GO, CNTs, GO/FCNTs mixture, and GNFG on the hydration process of the cement paste at an early stage (72 h) [60]. According to the w/c ratio, the cement, nanomaterials, SP, and water were mixed to a total mass of 5 g. The aqueous solution containing the nanomaterials was sonicated before mixing with the cement.

## 3. Results and Discussion

### 3.1. Characterization of the Graphite, GO, CNTs, FCNTs, GO/FCNTs Mixture, and GNFG

#### 3.1.1. Morphology Investigation

**Figure 3:**
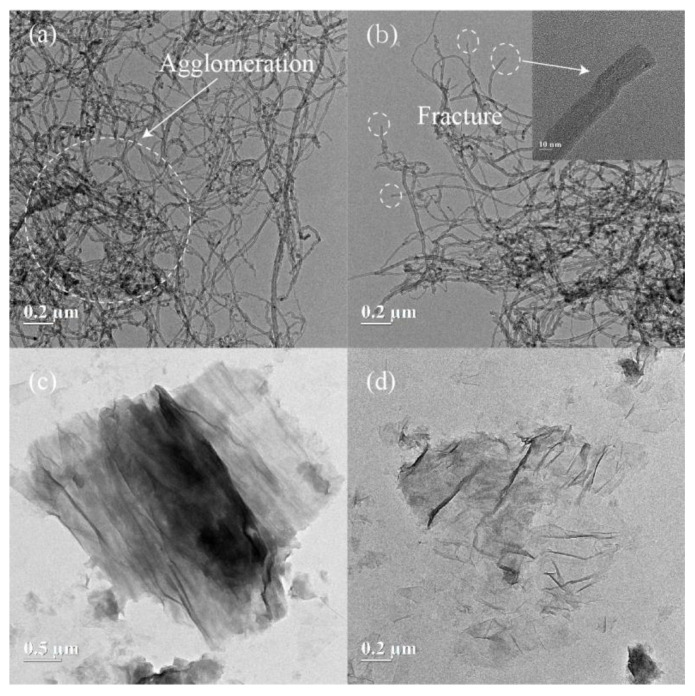
TEM images of the (**a**) CNTs, (**b**) FCNTs, (**c**) graphite, and (**d**) GO.

**Figure 4:**
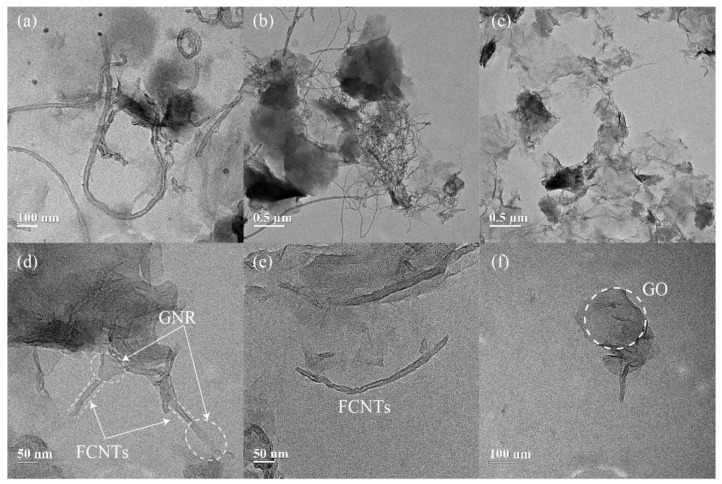
TEM images of the (**a**,**b**) GO/FCNTs mixture and (**c**–**f**) GNFG.

The TEM images of the graphite, GO, CNTs, FCNTs, GO/FCNTs mixture, and synthesized GNFG are shown in Figure 3 and Figure 4. As shown in Figure 3a, CNTs with high aspect ratios are agglomerated due to severe entanglement of CNTs and the absence of hydrophilic functional groups in the structure, which reduced their dispersion in aqueous solutions [61]. Figure 3b shows the FCNTs morphology; the FCNTs are fractured at the ends and do not have closed caps. This is due to the oxidation of the CNTs [62]. Meanwhile, the degree of agglomeration of FCNTs remains relatively high [63]. Figure 3c shows the morphological characteristics of graphite, indicating a multilayer graphite structure with a darker color. Figure 3d shows that the surface morphology of GO is wrinkled, like crumpled paper [64].

Figure 4a,b display the TEM images of the GO/FCNTs mixture, in which the GO and FCNTs were directly mixed without any chemical treatment. The results show that FCNTs are still being intertwined, with the GO and FCNTs remaining independent. Figure 4c depicts the overall morphological characteristics of GNFG. The FCNTs in the GNFG exhibit a shorter length, and the agglomeration degree is reduced compared with the FCNTs in the GO/FCNTs mixture. Pure CNTs are easily oxidized to FCNTs by strong oxidizing agents, such as sulfuric acid, nitric acid, and potassium permanganate [41,65]. In the synthesis of GNFG, sulfuric acid and potassium permanganate were also used; thus, FCNTs were generated. As the length of the FCNTs in GNFG shortens, the high aspect ratio decreases, reducing the entanglement and agglomeration in water.

**Figure 5:**
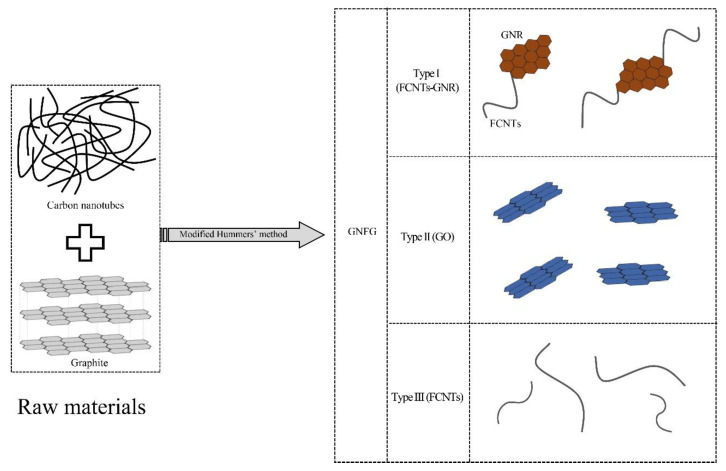
Schematic of the GNFG components.

Furthermore, as previously reported, when the amount of H_2_SO_4_ and KMnO_4_ was sufficiently high, CNTs could unzip and become graphene-oxide nanoribbons (GNR) [66]. However, in the synthesis process of GNFG, the amounts of H_2_SO_4_ and KMnO_4_ present are not enough to result in all CNTs unzipping to form GNR. Only part of the CNTs is unzipped into GNR, and the other part is oxidized to FCNTs, resulting in the formation of the unique connected structure of functionalized carbon nanotubes-graphene oxide nanoribbons (FCNTs–GNR), and the novel connected structure presented in Figure 4d. There are significant differences in the structures of GNR and FCNTs. As shown in Figure 4a,b, the walls of the FCNTs can be clearly seen. Unlike the FCNTs, the GNR in FCNTs–GNR does not have multiple layers of walls. In addition to this connected structure of FCNTs–GNR in GNFG, independently existing FCNTs and GO can also be found (Figure 4e,f). In summary, there are three types of components in GNFG: type I is FCNTs–GNR, type II is GO, and type III is FCNTs. To facilitate an interpretation of the images shown in Figure 4d–f, a schematic of the different components of GNFG is shown in Figure 5. The dispersion performance of this novel structure may be improved because the better dispersion of GNR can restrain the strong van der Waals forces of FCNTs connected to it.

#### 3.1.2. Determination of Crystal Structure

**Figure 6:**
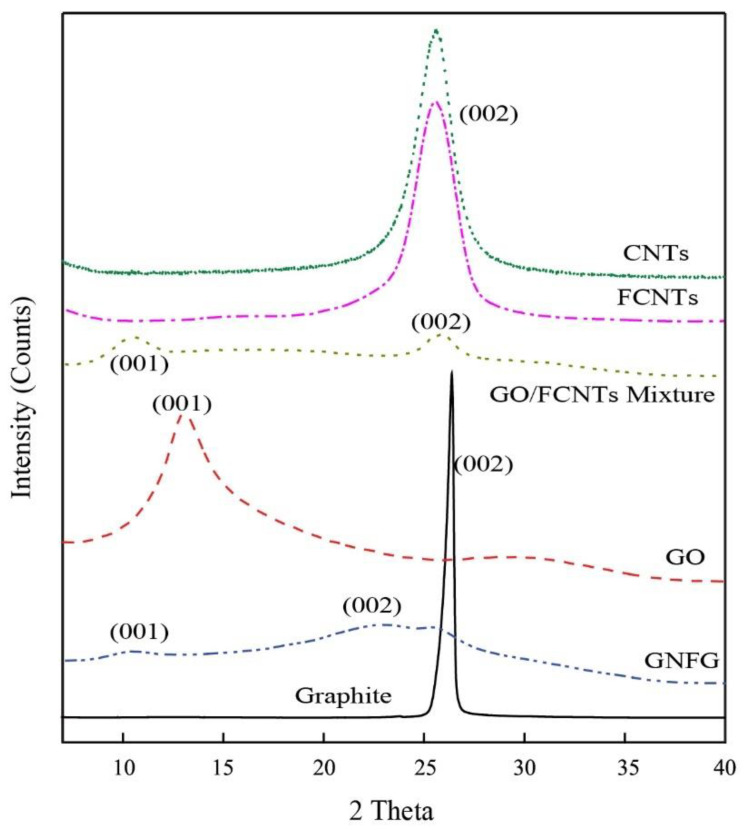
XRD patterns of the graphite, GO, CNTs, FCNTs, GO/FCNTs mixture, and GNFG.

The XRD patterns of graphite, GO, CNTs, FCNTs, GO/FCNTs mixture, and GNFG are presented in Figure 6. For graphite, a strong diffraction peak is observed at 2θ = 26.38° for the (002) graphitic plane, corresponding to an interlayer spacing of 0.337 nm (calculated using Bragg’s equation). However, in the case of GO, which obtained by the chemical treatment of graphite, the XRD peak is observed at a different position. Compared with graphite, the diffraction peak at 2θ = 26.38° disappeared, and a new enlarged peak appeared at approximately 2θ = 13.1° (interlayer spacing 0.675 nm) for the (001) crystal plane. This indicated that the crystal structure changed after the oxidization of graphite, resulting in an increased interlayer spacing. Sharma [47] suggested that an increase in the interlayer spacing was due to the insertion of oxygen-containing functional groups into the carbon atom layer. For the CNTs and FCNTs, a strong diffraction peak at 2θ = 25.58° is observed for the (002) plane with an interlayer spacing of 0.347 nm. The FCNTs, as the oxidation product of the CNTs, did not exhibit an altered structure compared to the CNTs; therefore, there were no significant differences in the XRD patterns. The XRD pattern of GNFG shows two small peaks, a (001) diffraction peak at 2θ = 10.17° with an interlayer spacing of 0.868 nm and a (002) diffraction peak at 2θ = 22.82° corresponding to an interlayer spacing of 0.389 nm. This diffuse peak at 2θ = 22.82° is also similar to the previous study [67]. To highlight the difference in GNFG, the XRD pattern of the GO/FCNTs mixture is also shown. For the GO/FCNTs mixture, unique diffraction peaks were preserved. The (002) diffraction peak of the FCNTs appears at 2θ = 25.58°; however, the (001) diffraction peak of GO is shifted to the left and appears at 2θ = 10.32°. This indicates that the interlayer spacing of GO in the GO/FCNTs mixture increased. Wang [68] reported that CNTs can be dispersed relatively well between GO sheets through uniform mixing of GO/CNTs, resulting in an increase in the GO layer spacing. Besides the differences in their crystal structures, there are some other differences based on their chemical functional groups. Thus, the functional groups of these materials were analyzed by FTIR and were discussed in detail in the next section.

#### 3.1.3. FTIR Spectroscopy

**Figure 7:**
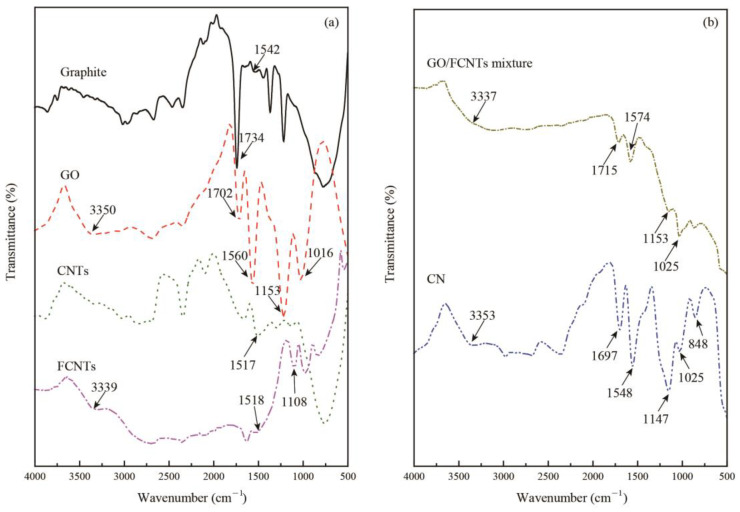
FTIR spectra: (**a**) graphite, GO, CNTs, and FCNTs, (**b**) GO/FCNTs mixture and GNFG.

The FTIR spectra of the graphite, GO, CNTs, FCNTs, GO/FCNTs mixture, and GNFG is shown in Figure 7a,b. There is a small peak arising from the C=C stretching vibration of graphite at 1542 cm^−1^ in the graphite spectrum [47,69]. In addition, owing to the purity of the graphite, there is a C=O stretching vibration peak at approximately 1734 cm^−1^ [47,69]. However, there are various functional groups in the GO structure after the graphite-oxidation treatment, including peaks for the –OH group at 3350 cm^−1^ and the C=O at 1702 cm^−1^ [47]. Moreover, an absorption peak for the C=C stretch is observed at approximately 1560 cm^−1^ [47,69]. The peaks at 1153 and 1016 cm^−1^ correspond to the stretching vibrations of C–O–C and C–O, respectively [47,69]. Since the functional groups were inserted into the graphite atomic layer, the layer spacing of GO increased; this was also evidenced by XRD results. Furthermore, a peak is observed in the CNTs spectra at 1517 cm^−1^, which is related to aromatic C=C bonds [47,69]. After oxidation, although the XRD patterns do not differ significantly, the functional groups of the FCNTs and CNTs are quite distinct. FCNTs have a C=C stretching vibration peak at 1518 cm^−1^, and –OH and C–O–C peaks at 3339 and 1108 cm^−1^, respectively. This indicates that after functionalization, oxygen-containing functional groups were inserted into the structure of the CNTs [47,69]. As for the GO/FCNTs mixture, the FTIR spectrum is similar to GO. The similarity in the spectrum is possibly attributed to the presence of GO in the GO/FCNTs mixture. In the case of GNFG, the peak appears at approximately 3353 cm^−1^, which corresponds to –OH stretching vibrations. Moreover, the peaks observed at 1697, 1548, 1147, 1025, and 848 cm^−1^ are related to the stretching vibrations of C=O, C=C, C–O–C, C–O, and epoxy groups, respectively [47,69]. Due to the graphene oxide nanoribbons in the GNFG complex, the FTIR spectrum of GNFG is also similar to the pure graphene oxide nanoribbons [70]. The properties of the functional groups could also determine the dispersion and stability of the nanomaterials in aqueous solutions, as determined via UV–vis spectroscopy.

#### 3.1.4. Dispersion and Stability of Nanomaterials

**Figure 8:**
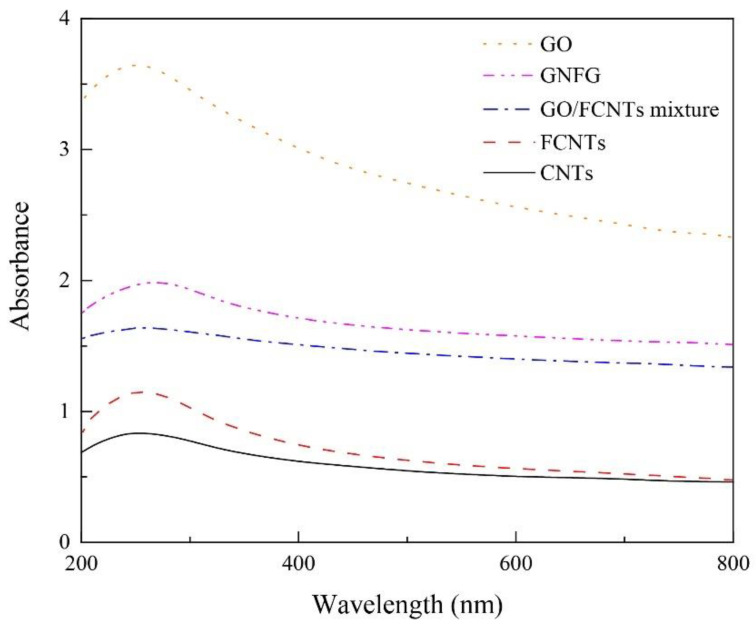
UV–vis spectra of the GO, CNTs, FCNTs, GO/FCNTs mixture, and GNFG.

To investigate the dispersion properties of GNFG in aqueous solutions, UV–vis spectroscopy was conducted on GNFG and the obtained results were compared to those of the CNTs, FCNTs, GO, and GO/FCNTs mixture (Figure 8). The absorbance is proportional to the dispersion of the nanoparticles because only uniformly dispersed nanoparticles can effectively absorb light in the UV–vis region [34]. Thus, the best dispersity in aqueous solutions was observed for GO, followed by GNFG, GO/FCNTs mixture, FCNTs, and CNTs. Owing to the high aspect ratio and strong van der Waals forces between the molecules, CNTs are likely to agglomerate in aqueous solutions and have poor dispersion [71]. In comparison with CNTs, the insertion of oxygen-containing functional groups results in the surface of the FCNTs being negatively charged due to the ionization, which generates electrostatic repulsion leading to improved dispersion of FCNTs in aqueous solutions [72]. As indicated in the FTIR spectra, GO contains hydrophilic oxygen-containing functional groups (–OH, –COOH), which generate electrostatic repulsion, and there is no strong van der Waals forces between the GO, resulting in the best dispersion [73]. Additionally, the absorbance of the GO/FCNTs mixture is higher than those of the CNTs and FCNTs, also due to the fact that the negatively charged surfaces of the FCNTs and GO generate electrostatic repulsion, increasing the degree of dispersion [34]. However, it should be noted that the absorbance of GNFG as a complex was higher than that of the GO/FCNTs mixture. The excellent dispersion of GNFG is attributed to several characteristics. Firstly, as described in the TEM results, due to the partial unzipping of CNTs to form FCNTs–GNR, the electrostatic repulsion on the GNR surface is able to inhibit the van der Waals forces between FCNTs in FCNTs–GNR. In particular, as the length of independently-existing FCNTs in GNFG shortens and the high aspect ratio decreases, the degree of intertwining is reduced, which results in an improved dispersion of GNFG [74]. Furthermore, the electrostatic repulsion generated by the negatively charged functional groups on the surface of independently existing GO and FCNTs can effectively improve the dispersion [75,76].

**Figure 9:**
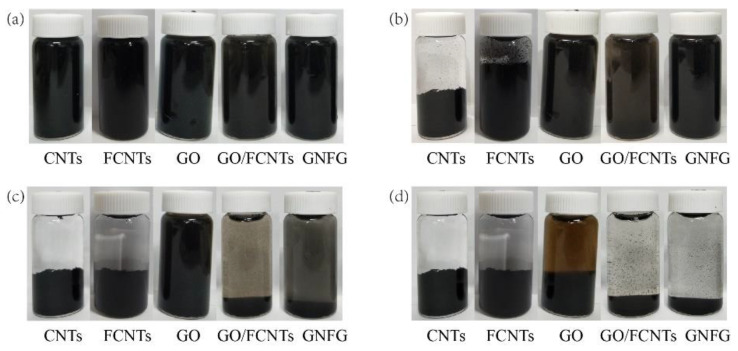
Dispersion of the nanomaterials over time after the ultrasonic treatment: (**a**) 0 min, (**b**) 10 min, (**c**) 6 h, and (**d**) 24 h.

To further evaluate the dispersion of nanomaterials in aqueous solutions, the stability of dispersion is also investigated. The dispersion stability of the CNTs, FCNTs, GO, GO/FCNTs mixture, and GNFG in aqueous solutions was determined over time after ultrasonic treatment, as shown in Figure 9. Figure 9a shows the results of observations made immediately after the ultrasonication, at which point all the nanomaterials were uniformly dispersed in the solutions and there were no clear agglomerates that precipitated out. After ten minutes (Figure 9b), the CNTs and FCNTs exhibited a different extent of agglomeration and precipitation. Although the absorbance of FCNTs in the UV–vis was higher than that of the CNTs, the stability of FCNTs in an aqueous solution was still unsatisfactory. However, the GO/FCNTs mixture, GO, and GNFG were still uniformly dispersed in the aqueous solution. After 6 h, the FCNTs and CNTs were completely agglomerated and precipitated out of the solution (Figure 9c). Furthermore, the color of the solution containing the GO/FCNTs mixture became lighter, indicating that the mixture of GO/FCNTs almost completely precipitated out of the solution. The GNFG remained partially and steadily dispersed in the solution, while GO remained steadily dispersed. After 24 h, only GO was still partially dispersed in the aqueous solution.

Although the preparation of the GO/FCNTs mixture is simple and the dispersion of the GO/FCNTs mixture is better than that of the CNTs and FCNTs, it is still less stable. However, chemically-treated GNFG not only has better dispersion in aqueous solutions, but also has a better stability. This also provided a guarantee that GNFG would show a reinforcement effect in cement-based materials. The advantages of GNFG as a reinforcing agent for cementitious systems will be further discussed in the following sections.

### 3.2. Mechanical Properties

**Figure 10:**
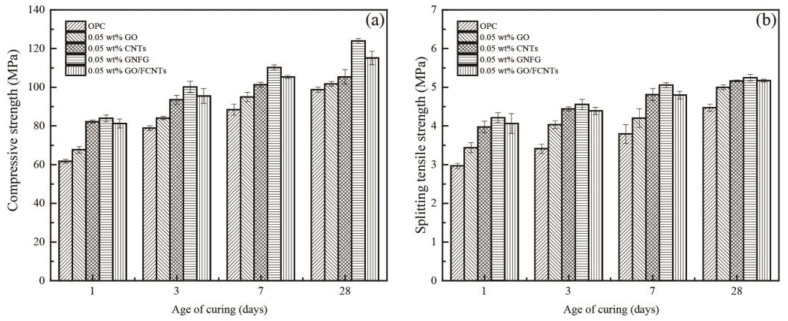
(**a**) Compressive and (**b**) splitting tensile strength of the specimens.

The effects of different nanomaterials (GO, CNTs, GO/FCNTs mixture, and GNFG) on the mechanical properties of hardened cement paste were assessed after 1, 3, 7, and 28 days. Figure 10a,b represent the compressive and splitting tensile strength of the cement paste with and without GO, CNTs, GO/FCNTs mixture, and GNFG, respectively. In comparison with OPC, after 28 days of curing, it was evidenced that incorporating 0.05 wt.% CNTs led to an increase in both the compressive strength (6.65%) and splitting tensile strength (15.39%) of the cement paste. Moreover, the addition of 0.05 wt.% GO increased the compressive and splitting tensile strengths of the cement paste by 2.98% and 11.76%, respectively, after 28 days of curing. This indicates that CNTs and GO can improve the mechanical properties of cement paste, which agrees with previous reports [77]. Addition of CNTs and GO increase the mechanical strength of the cement paste based on the following effects: (1) the filling effect, in which the CNTs and GO function as fillers in the nanoscale pores of the cement matrix; (2) the bridging effect, in which the nanomaterials connect the microcracks in the cement and inhibit the development of cracks; and (3) the nucleation effect, in which the nanomaterials provide more nucleation sites for hydration of the cement paste [30,78,79]. Further, when 0.05 wt.% of the GO/FCNTs mixture was added to the cement paste, the compressive and splitting tensile strengths were improved by 16.5% and 15.6%, respectively, fully demonstrating the synergistic effect of GO and FCNTs, as previously reported [34].

Notably, after hydration for 28 days, the 0.05 wt.% GNFG specimen showed the highest compressive strength (123.87 MPa) and splitting tensile strength (5.25 MPa), which were improved by 25.39% and 17.31%, respectively, compared to OPC. GNFG appeared to improve the mechanical properties of the cement paste, possibly because of the good dispersion and unique connected structure. Although GO has better dispersion in aqueous solutions than GNFG, the co-effect of GNFG can significantly improve the mechanical properties of the cement paste.

### 3.3. Microstructure Observations

**Figure 11:**
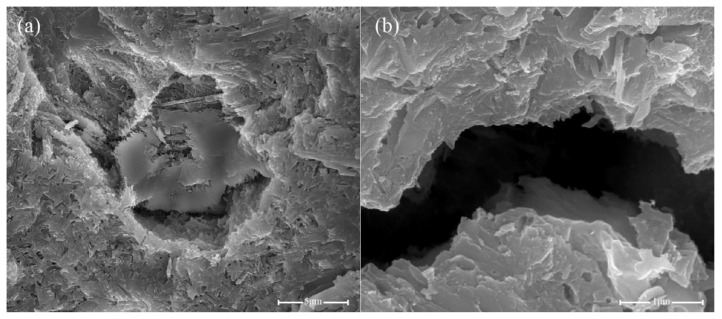
SEM images of OPC (**a**,**b**).

A previous study [80] showed that the mechanical strength of nanomaterial-incorporated hardened cement paste is related to its microstructure. Therefore, the microstructure of the fractured surface of hardened cement paste with and without nanomaterials, after 28 days of curing, was observed via SEM. Figure 11a,b show the SEM images of OPC as the control sample. For the cement composites without any nanomaterials, the cement matrix structure is not compact and porous, with several microscale pores and cracks that lead to relatively poor mechanical properties for OPC compared to nanomaterial-incorporated cement pastes.

**Figure 12:**
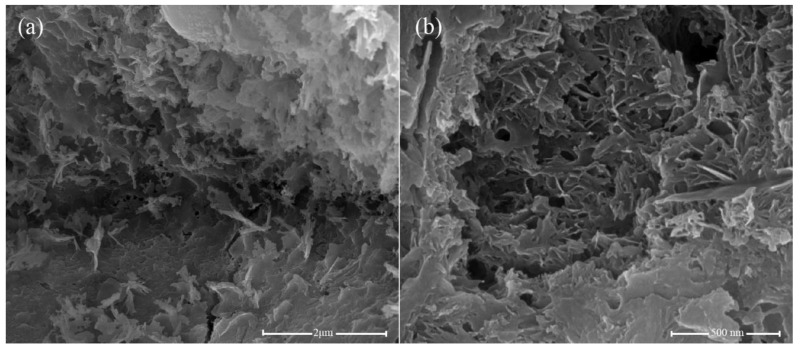
SEM images of 0.05 wt.% GO (**a**,**b**).

**Figure 13:**
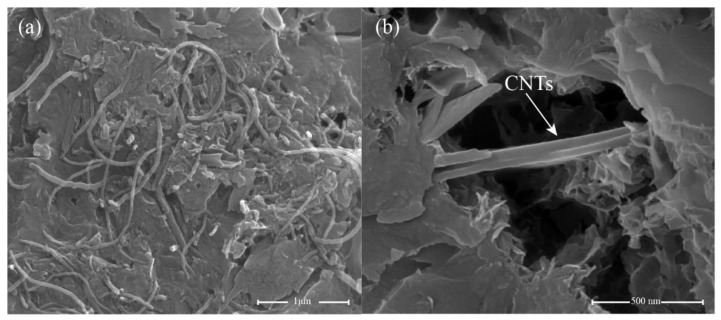
SEM images of 0.05 wt.% CNTs (**a**,**b**).

**Figure 14:**
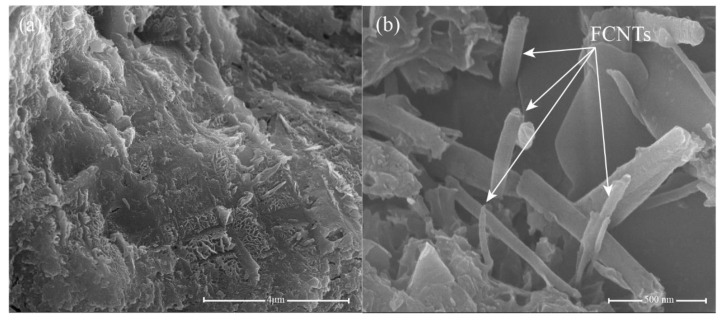
SEM images of 0.05 wt.% GO/FCNTs (**a**,**b**).

Compared to OPC, the microstructure of the 0.05 wt.% GO specimen (Figure 12a,b) is relatively compact, with reduced microscale cracks. GO can fill the microscale pores and cracks and provide more nucleation sites for cement hydration, thereby improving the microstructure of the cement. The addition of 0.05 wt.% CNTs resulted in a more uniform and compact structure, as the CNTs generated bridges between the cement hydrates, as shown in Figure 13. Previous studies [81,82,83] have already confirmed that the bridging effect of CNTs improves the load-bearing capacity of the cement matrix. In contrast, CNTs also show outstanding filling effects; they can fill the microscale pores and increase the compactness of the cement matrix. However, although CNTs can improve the microstructure of the cement, CNTs tend to agglomerate in the cement matrix (Figure 13a), which could interfere with its excellent mechanical properties. In addition, Figure 14a,b present the microstructure of cement paste with 0.05 wt.% GO/FCNTs mixture. It can be seen that the cement matrix of 0.05 wt.% GO/FCNTs mixture is compact, and some hydration products are covered on the surface of the cement matrix. Besides, the FCNTs exhibit a lower degree of agglomeration and are filled in the cement matrix.

**Figure 15:**
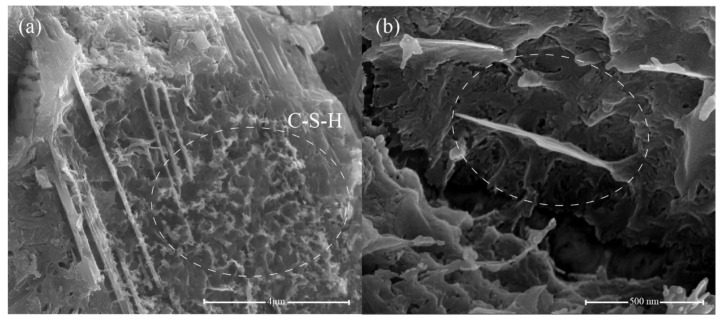
SEM images of 0.05 wt.% GNFG (**a**,**b**).

The microstructures of the cement hydrates containing 0.05 wt.% GNFG after 28 days of hydration are shown in Figure 15. It is clear that hydration products of calcium–silicate–hydrate (C–S–H) are uniformly distributed in the matrix without any large pores, as shown in Figure 15a. Moreover, in the 0.05 wt.% GNFG specimen, aggregation of FCNTs is not observed in the cement matrix. The GNFG can fill the microscale pores in the cement matrix and play a bridging role (Figure 15b). In addition, GNFG is able to bridge surficial Ca^2+^ of the cement matrix due to its wrinkled surface with oxygen-containing functional groups which enhances the interfacial bonding [84]. This also contributes to the very dense microstructure of cement paste and the 0.05 wt.% GNFG-incorporated specimen exhibiting the highest compressive strength and splitting tensile strength among the five groups.

### 3.4. Heat of Hydration

**Figure 16:**
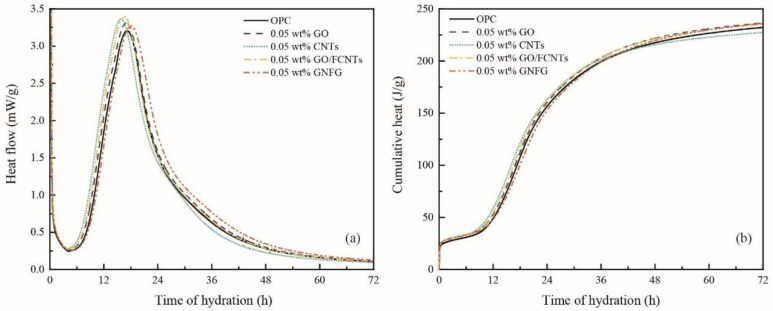
(**a**) Heat flow curves and (**b**) cumulative heat curves of the cement paste specimens.

Nanomaterials have a significant effect on the early hydration process of cementitious systems [85]. To fully observe and compare the effects of the different carbon nanomaterials on the early hydration process (within 72 h) of cement, isothermal calorimetry was used to evaluate the amount of heat released from the OPC and cement pastes containing 0.05 wt.% GO, CNTs, GO/FCNTs mixture, and GNFG (Figure 16a,b). Typically, exothermic reactions with four stages can be observed during the hydration process of the cement: the first stage is the initial period, the second stage is the induction period, the third stage is the acceleration period, and the fourth stage is the deceleration period [86,87]. Previous studies have shown that the SP delays the induction period [88]. The SP, as an anionic surfactant, will be more easily adsorbed on the surface of an oppositely charged cement. Thus, due to adding the SP to the cement paste, the surface of the cement particles was covered, which hindered the exchange of ions in the hydration system and reduced the hydration rate. In addition, the interaction between Ca^2+^ and the SP reduces the concentration of Ca^2+^, and hinders the nucleation of hydration products [89,90]. However, the five groups of specimens in this study contain the same content of SP, the delayed effect of SP on the early induction period of hydration can be ignored.

As shown in Figure 16a, although the addition of different nanomaterials, there was no significant influence on the initial and induction periods of the cement hydration process. However, from about four hours, the hydration reaction enters an acceleration period. It can be clearly observed that the maximum heat flow of the cement paste with nanomaterials is higher than that of OPC. Therefore, these carbon nanomaterials contribute obviously to the hydration of cement, which is also consistent with previous studies [91]. When all samples entered the deceleration period, it was found that the cement paste incorporating GNFG had a higher heat flow during the deceleration period compared to the other samples. Although the maximum heat flow of GNFG is slightly lower than that of specimens with other nanomaterials, the higher heat flow during the deceleration period results in a cumulative heat of 72 h that is not significantly different from that of specimens with other nanomaterials. In addition, it could also be found that the heat flow of the samples containing 0.05 wt.% CNTs was lower than all samples in the deceleration period and resulted in the lowest cumulative heat over 72 h (Figure 16b). This is attributed to the agglomeration of CNTs in the cement matrix as presented in the SEM image (Figure 13a), which has an impact on the deceleration period of the hydration reaction. Overall, owing to the addition of the nanomaterials (besides CNTs), the cumulative heat of the cement hydration increased after 72 h, indicating the enhanced degree of hydration of the cement at early stage. However, although CNTs promote the hydration reaction during the acceleration period, they also have some impact on the hydration reaction during the deceleration period. It is difficult to evaluate the degree of hydration of different samples at various periods by only using calorimetry. Therefore, XRD and TG analysis also were applied to investigate the effects of the CNTs, GO, GO/FCNTs mixture, and GNFG on the hydration reaction of cement pastes at various curing times.

### 3.5. XRD Analysis of the Hydration Products

**Figure 17:**
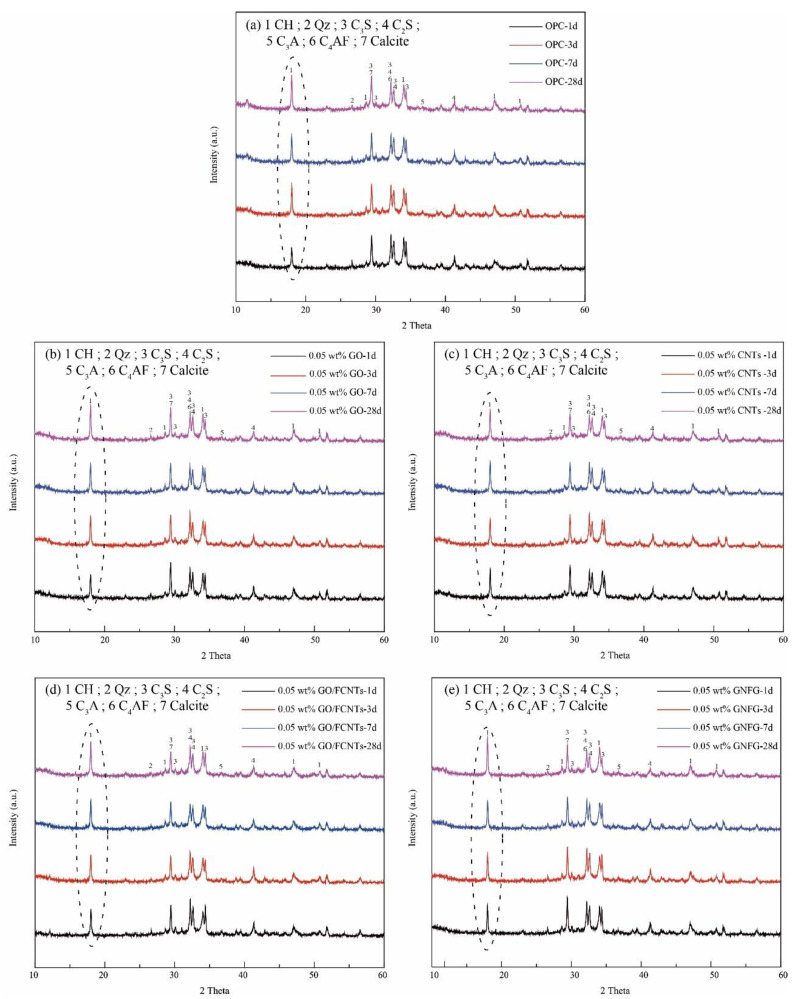
XRD spectra of (**a**) OPC, (**b**) 0.05 wt.% GO, (**c**) 0.05 wt.% CNTs, (**d**) 0.05 wt.% GO/FCNTs. and (**e**) 0.05 wt.% GNFG.

XRD was employed to qualitative analyze the hydration products and the consumption rate of the anhydrous phases at different hydration periods [92]. Previous research indicated that owing to the large specific surface area of CNTs and GO, more nucleation sites are present for cement hydration and more hydration products are generated [10,93]. The XRD patterns of OPC and the cement pastes containing 0.05 wt.% GO, 0.05 wt.% CNTs, 0.05 wt.% GO/FCNTs mixture, and 0.05 wt.% GNFG are shown in Figure 17. Usually, as the hydration reaction progresses, the unhydrated calcium silicate content gradually decreases [94]. As reported previously [95,96,97], the peaks of C_3_S and C_2_S overlap with those of the other minerals (C–S–H, calcium hydroxide (CH), and calcite). In Figure 17, the peaks of C_3_S and C_2_S also appear to overlap; thus, it was not easy to determine the changes in reduction.

It is well known that C–S–H and CH are produced simultaneously by the hydration of calcium silicate [47,98]. The relative hydration rate can be established by measuring the content of C–S–H or CH. In general, XRD is used to determine the CH content instead of the C–S–H content because CH has a hexagonal crystal structure that can be easily detected by XRD. In contrast, the C–S–H phase forms a semi-crystalline or amorphous phase, which limits the use of XRD as a detection technique [47,99,100,101]. In the crystallization patterns (Figure 17), the diffraction peaks of CH were observed at 2θ = 17.9°, 28.6°, 34.1°, 47.1°, and 50.1° [47]. For the OPC, 0.05 wt.% GO, 0.05 wt.% CNTs, 0.05 wt.% GO/FCNTs mixture, and 0.05 wt.% GNFG specimens, the CH diffraction peaks show significant differences. The XRD patterns of the five groups show that the 0.05 wt.% GNFG specimen has the highest CH intensity over the different periods, which indicates that GNFG improves the degree of hydration of cement paste and more hydration products are produced compared to OPC. In addition, when 0.05 wt.% CNTs, GO, and GO/FCNTs mixture were added to the cement, the CH content also increased. This indicates that hydration was promoted when these nanomaterials were used. The amounts of CH in each sample at the different periods were quantified by TG analysis.

### 3.6. Thermogravimetric Analysis

**Figure 18:**
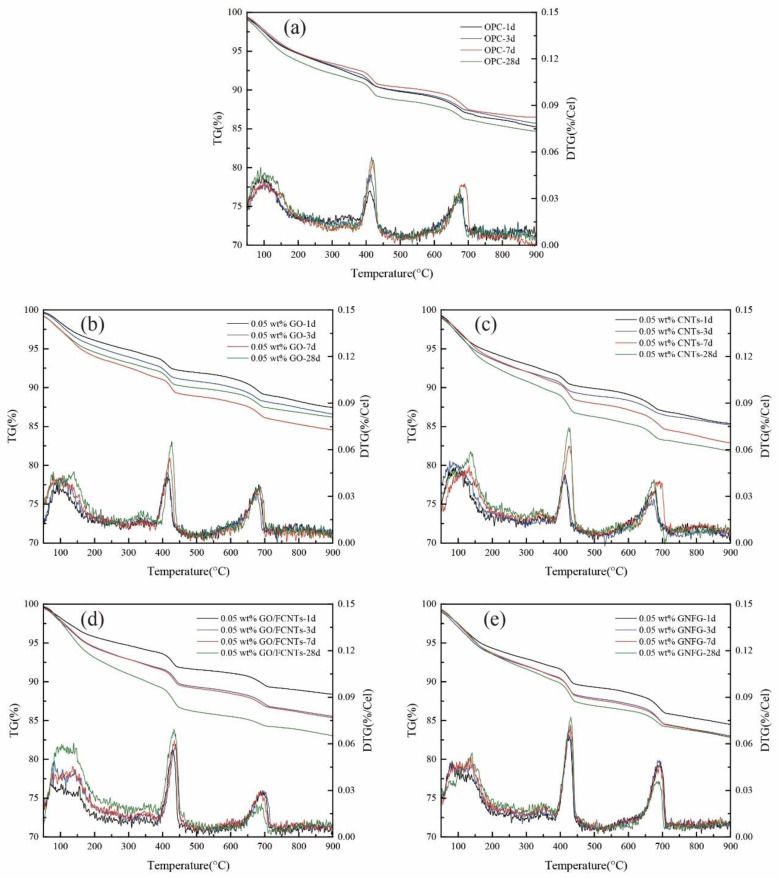
TG/DTG curves of the samples containing (**a**) OPC, (**b**) 0.05 wt.% GO, (**c**) 0.05 wt.% CNTs, (**d**) 0.05 wt.% GO/FCNTs, and (**e**) 0.05 wt.% GNFG.

The OPC, 0.05 wt.% GO, 0.05 wt.% CNTs, 0.05 wt.% GO/FCNTs mixture, and 0.05 wt.% GNFG samples were analyzed by TG and DTG at 1, 3, 7, and 28 days, as shown in Figure 18. The mass loss at approximately 100 °C is related to the evaporation of free water. Mass loss in the range of 400–500 °C is due to the decomposition of CH, and between 600 and 700 °C is attributed to calcium carbonate (CaCO_3_) decomposition [102]. However, as stated in the XRD section, calcium silicates produce both C–S–H and CH when hydrated [47]. Based on the values in Figure 18, the CH content for each sample at the different curing times was calculated according to Equation (1). ${\mathrm{WL}}_{\mathrm{Ca}{\left(\mathrm{OH}\right)}_{2}}$ represents the percentage weight loss of CH, and $m_{\mathrm{Ca}{\left(\mathrm{OH}\right)}_{2}}$ and $m_{H_{2}O}$ are the molecular masses of portlandite (74 g/mol) and water (18 g/mol), respectively. The calculation results are listed in Table 6.

(1) $$\mathrm{Ca}{\left(\mathrm{OH}\right)}_{2,\mathrm{measured}}={\mathrm{WL}}_{\mathrm{Ca}{\left(\mathrm{OH}\right)}_{2}}\times m_{\mathrm{Ca}{\left(\mathrm{OH}\right)}_{2}}/m_{H_{2}O}.$$

As shown in Figure 18 and Table 6, compared with the samples containing 0.05 wt.% CNTs, GO, GO/FCNTs mixture, and GNFG, OPC showed the lowest relative amount of CH (1 d: 5.43%; 3 d: 6.42%; 7 d: 7.23%; and 28 d: 7.44%) for each period. This indicates that OPC has fewer hydration products and shows a lower degree of hydration, which is also consistent with the XRD results. For the samples with nanomaterials, the hydration of the cement paste was enhanced at each curing time, although the degree of enhancement differed. Using a dosage of 0.05 wt.%, GNFG induces the greatest enhancement in hydration among the four nanomaterials, followed by the GO/FCNTs mixture, CNTs, GO, and OPC. This also proves that GNFG can promote the hydration of cement paste and generate more hydration products, and the promotion effect is better than other nanomaterials.

## 4. Conclusions

In this study, the highly-dispersed GNFG was hydrothermally synthesized and its effects on the mechanical properties, hydration kinetics, hydration products, and microstructure of cementitious composites were investigated. The results were compared with those using the composites incorporating other typical carbon nanomaterials. Based on the experimental results, the following conclusions can be drawn:

(1) GNFG, as a new carbon nanomaterial, was successfully synthesized via a chemical method, and there are three different components (FCNTs–GNR, FCNTs, and GO) of GNFG due to the treatment conditions and led to better dispersibility.
(2) Although the GO, CNTs, GO/FCNTs mixture, and GNFG improved the compressive and splitting tensile strength of cement paste at the same content (0.05 wt.%), GNFG induced the greatest improvement in the mechanical properties of the cement paste due to the denser microstructure.
(3) GNFG, as a new reinforcing nanomaterial for cementitious systems, similar to other nanomaterials, can fill cracks and play a bridging role in the cement matrix.
(4) GNFG can increase the maximum heat flow and cumulative heat of cement hydration reaction, promote the hydration reaction and generate more hydration products.

These experimental results demonstrate that GNFG exhibits excellent potential as a new nano-reinforcing agent for cementitious materials, especially relating to the improvement in the mechanical properties of cementitious composites.

## Figures and Tables

**Table 1:** Chemical composition of OPC.

Chemical Composition	SiO_2_	Al_2_O_3_	Fe_2_O_3_	CaO	MgO	K_2_O	SO_3_	TiO_2_	LOI	Total
(wt.%)	18.43	2.83	2.17	68.17	2.37	1.11	3.03	0.15	1.72	100

**Table 2:** Properties of the CNTs.

Outside Diameter (nm)	Inside Dimeter (nm)	Length (μm)	Ash (%)	Purity (%)	SSA (m^2^/g)	Color
10–20	5–10	10–30	<1.5	95	>200	Black

SSA: specific surface area.

**Table 3:** Properties of graphite flakes.

Mesh	Purity (%)	Density (g/mL)	Boiling Point (°C)	Particle Size (µm)	Color
325	99	2.2	4830	<50	Black

**Table 4:** Properties of the polycarboxylate superplasticizer.

Water Content (%)	pH Value (10% Solution)	Active Component (%)	Bulk Density (kg/m^3^)
<3	6.0–8.0	>90	450

**Table 5:** Mix proportions.

Specimen	Cement (g)	Water (g)	SP (g)	GO (g)	CNTs (g)	GO/FCNTs (g)	GNFG (g)
OPC	100	30	0.1	–	–	–	–
0.05 wt.% GO	100	30	0.1	0.05	–	–	–
0.05 wt.% CNTs	100	30	0.1	–	0.05	–	–
0.05 wt.% GO/FCNTs	100	30	0.1	–	–	0.05	–
0.05 wt.% GNFG	100	30	0.1	–	–	–	0.05

**Table 6:** Percentage content (%) of Ca(OH)_2_ in the hardened cement paste after different curing times.

Specimen	Ca(OH)_2_ Content (%)			
	1 Day	3 Days	7 Days	28 Days
OPC	5.43%	6.42%	7.23%	7.44%
0.05 wt.% GO	6.17%	6.50%	7.46%	7.62%
0.05 wt.% CNTs	6.29%	6.81%	8.43%	10.03%
0.05 wt.% GO/FCNTs	6.83%	8.54%	9.35%	10.27%
0.05 wt.% GNFG	8.62%	9.49%	9.66%	10.97%

## Data Availability

Data are contained within the article.
